# Analysis of platelet rich plasma preparations by routine centrifuge versus two commercially available standardized centrifuges

**DOI:** 10.12669/pjms.42.8.15762

**Published:** 2026-08

**Authors:** Syed Kamran Ahmed, Asifa Abdul Hakeem, Sadaf Saeed

**Affiliations:** 1 Syed Kamran Ahmed, FCPS Department of Orthopaedics & Traumatology, Indus Hospital & Health Network, Karachi, Pakistan; 2 Asifa Abdul Hakeem, FCPS Department of Orthopaedics & Traumatology, Indus Hospital & Health Network, Karachi, Pakistan; 3 Sadaf Saeed, FCPS Plastic surgery Department of Plastic Surgery, Indus Hospital & Health Network, Karachi, Pakistan

**Keywords:** Blood Platelets, Centrifugation, Leukocytes, Platelet Count, Platelet-Rich Plasma

## Abstract

**Background & Objective:**

Platelet-rich plasma (PRP) therapy is increasingly used in regenerative medicine, orthopedics, and dermatology; however, the lack of standardization in PRP preparation leads to variability in platelet yield and composition. This study aimed to compare the quality and efficiency of PRP prepared using a routine laboratory centrifuge with two commercially available standardized centrifuge systems.

**Methodology:**

This comparative cross-sectional study was conducted in the Department of Orthopedics & Traumatology and the Department of Plastic surgery at Indus Hospital & Health Network (IHHN), Karachi, over a period of six months from August, 2025 to January 2026. Non-probability consecutive sampling was employed. Thirty-six PRP samples were prepared using routine single-spin, routine double-spin, and two commercial centrifugation methods. Platelet and leukocyte counts, hemoglobin contamination, and platelet recovery were analyzed using SPSS version 26.

**Results:**

Commercial standardized centrifuge systems yielded significantly higher platelet concentrations (mean 835 ± 120 ×10^9^/L) compared to routine centrifuges (mean 25.5 ± 10.9 ×10^9^/L; p = 0.005). Platelet recovery and fold increase were also superior in commercial systems (mean 81.6% ± 5.94 vs. mean 10.65% ± 4.59). Leukocyte count and hemoglobin contamination were relatively lower in routine centrifuge methods compared to commercial PRP therapy. Among routine protocols, both single-spin and double-spin methods produced poor platelet counts when the entire supernatant fluid was examined as in our study.

**Conclusion:**

Commercial standardized centrifuges produce PRP with reliable and greater platelet enrichment. Routine centrifuge, on the other hand, consistently produced poor platelet counts in the entire supernatant fluid and hence the lower one third only should be used preferably by double spin method. Different spin protocols and collection methods need to be evaluated to better document platelet enhancement in routine centrifuge use.

***List of abbreviations:***

**PRP:** Platelet-Rich Plasma. **PPP**: Platelet Poor Plasma. **WBC:** White Blood Cells.

**RPM:** Revolutions Per Minute. **ACP:** Autologous Conditioned Plasma.

## INTRODUCTION

Platelet-rich plasma (PRP) therapy has emerged over the last two decades as a widely used autologous biologic for regenerative medicine, orthopedics, dermatology, dentistry and wound care.^1^ PRP is prepared from the patient’s own blood and concentrated to deliver a supraphysiologic number of platelets, which release growth factors (PDGF, TGF-β, VEGF, EGF and others) that modulate inflammation, angiogenesis and tissue repair.^2^

Its relative safety, autologous origin, and ease of preparation have driven exponential clinical adoption across specialties. However, despite extensive clinical use and numerous randomized trials, the literature reports highly variable outcomes, in part because PRP preparations differ markedly in platelet concentration, leukocyte content and activation status depending on the preparation technique.^3^ The fundamental step in all PRP preparations is differential centrifugation.^4^ Simple variables such as relative centrifugal force (RCF, ×g), spin duration, number of spins (single vs double), braking, and tube geometry significantly influence platelet yield, leukocyte carryover and growth factor content. The heterogeneity in the final product complicates the interpretation of clinical trials and may partly explain contradictory efficacy results across indications like osteoarthritis and tendon disorders.^5^,^6^

Differences between routine laboratory centrifuges and standardized devices have not been thoroughly compared, particularly for platelet and leukocyte counts in identical sample.^7^,^8^ Recent trials and reviews emphasize the need for proper product characterization, while meta-analyses report high variability due to inconsistent preparation and reporting methods.^9^ Lack of standardization risks combining biologically different products when data are pooled across studies.^10^

From a bench perspective, controlled methodological work has quantified how centrifugation parameters alter PRP composition. Lower RCF and shorter times can preserve platelet recovery but may leave more erythrocyte contamination; conversely, higher RCF tends to sediment erythrocytes and some leukocytes more effectively but may also force platelets into unwanted layers, reducing recovery.^10^ A study done by Harrison et al.^5^ showed that extracting platelets in plasma beginning ½ ml below the buffy coat and extending ½ ml above the buffy coat achieved a higher platelet count (mean 10.480 ±0.224 with p <0.001) compared to platelet extracted from plasma only at the beginning of the buffy coat(mean 9.090±0.219 with p<0.001).These mechanistic data argue that centrifuge type, difference in speed and time could produce clinically meaningful differences in PRP production and its efficacy.^11^ According to a study conducted by Ghanem et al.^12^ in patients of Androgenic alopecia treated with PRP, mean difference in hair density was 4.10% more in single versus double- spin.

Although PRP therapy has gained immense popularity across regenerative medicine, dentistry, orthopedics, and dermatology, the lack of standardization in its preparation remains a major barrier to consistent clinical outcomes. This study concept seeks to bridge that knowledge gap, providing evidence-based guidance for clinicians and researchers to select the most efficient, reliable, and cost-effective method for PRP preparation. The study aimed to compare the platelet yield, leukocyte concentration, and overall quality of PRP prepared using a routine laboratory centrifuge versus two commercially available standardized PRP centrifuge systems, to deter mine the reproducibility, efficiency, and suitability of each method for clinical applications.

Prior to conducting the main study, a pilot study was conducted on six consenting volunteers. Three samples from healthy volunteers were run on a routine Benchtop lab centrifuge Megafuge 8 series model, Megafuge 8 R, 230V on 4000 RPM, 2500 RPM and 1200 RPM. The fourth sample was spun in a commercially available Arthrex machine. Samples run in a routine benchtop lab centrifuge had markedly reduced platelet count, but samples run in commercially available Arthrex centrifuge showed a significant increase in platelet count.

Two samples were run in another widely used commercial centrifuge (Model 80-2, power 80W, power supply 220V 50Hz), one done in non-gel vacuum tube containing sodium citrate, and second one done in gel PRP tube. Results acquired from both tubes showed a significant decrease in platelet count. There was no difference between gel and non-gel tubes.

## METHODOLOGY

This comparative cross-sectional study was conducted at the Department of Orthopedics & Traumatology and Department of Plastic surgery at Indus Hospital & Health Network (IHHN), Karachi, over a period of six months from August, 2025 to January 2026.

### Ethical approval:

It was granted by the Institutional Review Board of Indus Hospital and Health Network, Karachi, under approval number: HHN_IRB_2025_02_001; dated August 15, 2025.

The sample size was calculated using OpenEpi calculator. By keeping a 95% confidence level, 80% power, a ratio of 2:1, mean platelet count in routine centrifuge at 12.51 ± 5.90 and in commercial centrifuge at 7.25 ± 4.74, sample size came out to be 36, 24 from the routine centrifuge group and 12 from the commercial centrifuge groups.

The sampling technique employed was non-probability consecutive sampling. Healthy adult participants aged between 18 and 60 years, irrespective of gender, controlled diabetes , controlled hypertension, no chronic uncontrolled disease and bleeding disorder, having normal platelet count at baseline, who provided written informed consent were included in the study. Individuals with chronic medical conditions such as uncontrolled diabetes mellitus or hypertension, pregnancy, those with a recent history of blood transfusion, those having history of bleeding disorders or those taking antiplatelet medications such as aspirin or clopidogrel were excluded.

After ethical approval and informed consent, participants’ demographic information, including age, gender, weight, and medication status, was recorded. Blood samples were drawn under aseptic conditions by trained laboratory staff and processed immediately. PRP was prepared using six different centrifugation methods. A routine centrifuge is a commercially available device which applies centrifugal force to anticoagulated whole blood set manually and has not passed any rigorous testing which proves its efficacy in enhancing platelet count. For the routine centrifuge (Model 80-1, power 40W, power supply 220V 50Hz, PRP tubes ACD plus gel 10 mL), four spin protocols were tested: single spin at 4000 RPM for 10 minutes for six samples allocated as Group-1; single spin at 4000 RPM for 15 minutes for six samples allocated as Group-2; double spin at 2000 RPM for 10 minutes followed by 4000 RPM for 10 minutes for six samples allocated as group 3; double spin at 2000 RPM for 15 minutes followed by 4000 RPM for 15 minutes for six samples allocated as -4. In every group at the end of centrifugation, the entire supernatant fluid was sent for examination.

The commercially marketed systems are launched after rigorous testing of the product achieved as a result of a specified spin model (1500 RPM for Arthrex ACP system vs 4000RPM for HSC Neo PRP system) on a specific centrifuge of a high quality (whose spin models are reliable and reproducible without human errors). For the commercial systems, two standardized devices were used: for six samples the HSC Neo PRP System (Neogenesis, Korea) using 30 mL tubes centrifuged at 4000 RPM for five minutes allocated as Group-5 and the Arthrex ACP System (Arthrex Inc.) for six samples using 16 mL tubes centrifuged at 1500 RPM for five minutes allocated as Group-6, as per manufacturer protocols.

Each PRP sample was subsequently analyzed for platelet count, white blood cell (WBC) count, and hemoglobin contamination using an automated hematology analyzer. Platelet recovery percentage and platelet fold increase compared to baseline blood counts were calculated.

Arthrex kit produced two layers on centrifugation, around 6 cc upper clear layer all of which was extracted as PRP. The lower layer of concentrated RBCs and WBCs was left behind.

Neo HSC kit produced three visible layers. Upper clear layer around 8 cc very thin middle layer (Buffy coat) and a lower layer of concentrated RBCs. Only the lower three cc of upper layer including the buffy coat (middle layer) were extracted as PRP on the manufacturer recommendation.

PRP was defined as an autologous blood derivative containing a higher concentration of platelets than baseline whole blood, rich in growth factors and cytokines that promote healing. A centrifuge was defined as a laboratory device that separates blood components by density through high-speed rotation, concentrating platelets in the upper plasma layer. The “buffy coat” was identified as a thin, yellowish-white layer formed between plasma and red blood cells after centrifugation, rich in platelets and leukocytes. A healthy individual was defined as a volunteer free from acute or chronic diseases, both physically and mentally stable, at the time of sample collection.

At the end of the study four more samples were allocated to each group 1-4 (on routine centrifuge Model 80-1, power 40W, power supply 220V 50Hz) in gel tubes to decrease the bias between gel and non-gel tubes. However, since it was only one test per group , it was not included in the sample size. after completion of the sample size (Table-IV).

**Table-I:** Demographic Characteristics of Participants (n = 36).

Variable	Mean ± SD / Frequency (%)
Age (years)	34.8 ± 8.2
Weight (kg)	78.4 ± 4.6
***Medication use***	
None	34 (94.4%)
Mild analgesics	2 (5.6%)

**Table-II:** Platelet and WBC Counts in whole blood and PRP Prepared by different centrifugation methods.

Group	Centrifuge Type	Spin Protocol	Mean Platelet Count in Whole Blood at baseline (×10^9^/L)	Mean Platelet Count in PRP (×10^9^/L)	Platelet Fold change	Mean WBC Count in PRP (×10^9^/L)	Mean Hemoglobin Contamination (g/dL)
1	Routine	Single spin 4000 RPM × 10 min	240 ± 42	30.6 ± 10	7.9 times decrease	1.0 ± 0.9	0.9 ± 0.3
2	Routine	Single spin 4000 RPM × 15 min	238 ± 40	37.5 ± 5	6.3 times decrease	0.3 ± 0.2	0.4 ± 0.2
3	Routine	Double spin 2000/4000 RPM × 10 min each	242 ± 44	21.5 ± 10	11.3 times decrease	0.5 ± 0.2	0.3 ± 0.2
4	Routine	Double spin 2000/4000 RPM × 15 min each	240 ± 39	12.5 ± 10	19.2 times decrease	1.0 ± 0.6	0.5 ± 0.1
5	Commercial (Neo PRP – Neogenesis Korea)	4000 RPM × 5 min	243 ± 46	910 ± 140	3.7 times Increase	20.6 ± 0.4	2.01 ± 0.5
6	Commercial (Arthrex ACP – Arthrex Inc.)	1500 RPM × 5 min	239 ± 43	760 ± 130	3.2 times Increase	3.95 ± 0.3	3.95 ± 0.5

**Table-III:** Platelet Recovery and Efficiency of Each Centrifugation Method.

Group	Centrifuge Type	Platelet Recovery (%)	Platelet Loss (%)	WBC Reduction (%)
1	Routine (Group 1)	12.75	87.25	83.3
2	Routine (Group 2)	15.75	84.25	95
3	Routine (Group 3)	8.88	91.12	91.7
4	Routine (Group 4)	5.21	94.97	83.3
5	Commercial (Neo PRP)	83.7	16.3	-42
6	Commercial (Arthrex ACP)	79.5	20.5	43.6

**Table-IV:** Platelet and WBC Counts in Whole Blood and PRP Prepared by Single and double centrifuge methods in Non-Gel Tubes.

Group	Centrifuge Type	Spin Protocol	Platelet Count in Whole Blood (×10^9^/L)	Platelet Count in PRP (×10^9^/L)	Platelet Fold change	WBC Count in PRP (×10^9^/L)	Hemoglobin Contamination (g/dL)
1	Routine	Single spin 4000 RPM × 10 min	266	236	1.13 times decrease	0.2	0.1
2	Routine	Single spin 4000 RPM × 15 min	202	162	1.25 times decrease	0.2	0.2
3	Routine	Double spin 2000/4000 RPM × 10 min each	179	176	1.02 times decrease	0.2	0.1
4	Routine	Double spin 2000/4000 RPM × 15 min each	240	63	3.81 times decrease	0.1	0.1

Similarly, two tests was added to the HSC Neo PRP system to see platelet count difference between upper most and middle layer of PRP in HSC Neo PRP System (Neogenesis, Korea). These samples were also not included in sample size (Table-V).

**Table-V:** Platelet and WBC Counts in Whole Blood and upper most layer of Plasma prepared by HSC Neo PRP System (Neogenesis, Korea).

Group	Centrifuge Type	Spin Protocol	Platelet Count in Whole Blood (×10^9^/L)	Platelet Count in PRP (×10^9^/L)	Platelet Fold change	WBC Count in PRP (×10^9^/L)	Hemoglobin Contamination (g/dL)
1	Commercial NeoPRP	4000 RPM × 5 min	334	219	1.52 times decrease	0.1	0.1
2	Commercial NeoPRP	4000 RPM × 5 min	312	225	1.39 times decrease	0.1	0.1

### Statistical analyses:

All statistical analyses were performed using IBM SPSS version 26. Normality was tested using shapiro-wilk test. Quantitative variables such as age and weight were expressed as mean ± standard deviation (SD). Categorical variable such as medication status was presented as frequency and percentage. Mean and SD of platelet count increase, platelet fold change, WBC count, and mean hemoglobin contamination were calculated for routine and commercial centrifuges.

## RESULTS

A total of 36 healthy participants were enrolled in the study. The mean age of the participants was 34.8 ± 8.2 years, exclusively males. The average body weight was 78.4 ± 4.6 kg, reflecting a generally healthy adult population. Most participants, i.e., 34 (94.4%) were not taking any regular medications at the time of sample collection, ensuring that platelet or leukocyte counts were not influenced by external pharmacologic factors (Table-I).

The analysis of platelet and leukocyte counts revealed notable differences among the various centrifugation methods. Whole blood platelet counts ranged from 238×10^9^/L-240(×10^9^/L) across all groups, confirming baseline uniformity prior to processing. However, PRP prepared using commercial centrifuges (Neo PRP and Arthrex ACP) demonstrated higher mean platelet concentrations compared to routine centrifugation methods. The Neo PRP system achieved a high platelet yield (910 ± 140 × 10^9^/L), corresponding to 3.7 times increase, followed by Arthrex system (760 ± 130× 10^9^/L) with 3.2 times increase in platelet count**.** In contrast, single-spin and double-spin routine centrifuge methods showed decreased platelet yield, ranging between 6.3-19.2 times decrease. The mean WBC count and hemoglobin contamination in PRP were lowest in the routine centrifuge, while it was high in commercial standardized centrifuges (Table-II).

The efficiency analysis revealed that both commercial centrifuge systems outperformed routine centrifugation in terms of platelet recovery ( Table-III). Four additional samples collected in non-gel tubes, by all routine centrifuged methods, also led to significant decrease in platelet count, rather, platelet count was reduced in centrifuged samples (Table-IV). Two more samples run in the HSC Neo PRP System (Neogenesis, Korea) showed no increase in platelet count in upper most layer signifying it to be Platelet poor plasma as already described by the manufacturer (Table-V).

## DISCUSSION

PRP has been used by multiple medical specialties for a number of purposes. Reviews and trials report better outcomes with higher platelet concentrations highlighting the importance of reliable concentrates from various centrifuge systems and kits. Consistent higher platelet yields from commercial standardized systems translate into improved efficacy for many indications. A systematic evaluation emphasized the wide inter-study heterogeneity in PRP preparation and the need for standardized reporting (platelet counts, leukocyte counts, activation methods), reinforcing why head-to-head comparisons, such as ours, are important for understanding specific differences leading to clinical guidelines.^13^,^14^

In this study, commercially standardized PRP systems produced higher platelet concentrations, greater platelet recovery. The leucocyte count was somewhat higher in Neo PRP and mildly raised in Arthrex system. Hemoglobin concentration though higher in both might be result of blood tinge while processing the specimen as for said systems of both machines, non gel PRP tubes were available. The same can happen when non gel tubes are used in routine centrifuges as a result of human error during aspiration.

Our findings are similar to a recent study conducted by Srinivasan R et al. and Prysak MH et al.^15^,^16^ which reported high platelet counts in PRP formed by commercial standard centrifuges and low platelet counts in a routine centrifuge. However we studied the entire supernatant fluid and not the lower third, which lead to dilution of the specimen in routine centrifuge specimens.

Our study differed from a study done by Gupta V et al.^17^ because that study had slightly different technique and speed of spin. Their technique included first spin of 923 RPM for ten minutes (soft spin), followed by extraction of supernatant fluid. This supernatant fluid was subjected to another centrifugation cycle at 1460 RPM for ten minutes (hard spin) after which only the lower one third was extracted as PRP. The upper two third was discarded. From the above procedure two cc PRP was extracted from twenty cc of blood. In our study, we have done a double spin on the same tube using 2000 RPM for first spin cycle followed by a second spin at 4000 RPM without extracting supernatant fluid between spin cycles. Whole supernatant fluid was taken rather than lower one third for testing. This is not a standard way of harvest, however common practice for some surgeons and physicians, as otherwise the quantity of PRP will sharply decrease to 1.5-1.75 ml (lower one third of five cc supernatant produced from ten cc blood).

The study done by Saqlain et al.^11^ uses a double spin method comparative to Gupta et al.,^17^ however they used 1000 RPM for first soft spin and 800 RPM for the second hard spin. This is contrary to having a higher RPM for the second hard spin. This shows variations in the soft and hard spin cycles between various users. Still, they were able to produce platelet concentrate better in a double spin as compared to a single spin. They also aspirated the lower one third of the supernatant fluid in the end.

The Neo PRP HSC system produces three cc of PRP from thirty cc of blood (lower three cc of upper clear layer including a very thin buffy coat) If the upper five cc of the clear layer are used just to increase the volume of PRP it will lead to significant decrease in actual platelet concentration since the upper five cc is PPP and not PRP (Table V). This finding is similar to what happened in our standard centrifuge specimens.

This is in contrast to the Arthrex kit using a closed double syringe system, where six cc are produced as upper layer (all PRP) from fifteen cc blood. There was consistently high platelet count and it had completely closed double syringe system with the least risk of contamination. Therefore, we suggest it to be the best suited for joint injection where breach in sterility cannot be tolerated at all.

Few studies showed a higher platelet concentrations using well-tuned single-spin procedures or alternate tube chemistries.^18^ In our data, consistent with literature, carefully defined selection of spin parameters as in commercial centrifuges determined a consistent platelet yield^19^. Our study is consistent with a studies documenting that commercial and apheresis-level systems generally give higher percent platelet recovery than simple open tube single spin protocols.^20^,^21^ This comparison is for gel tubes. When we did it in non-gel tubes, platelet counts were slightly better than gel tubes, but no increase in counts to be labelled PRP.

### Strengths and Limitations:

The present study has certain strengths and limitations. It clearly supports the consistent delivery of concentrated platelets from two standardized commercial centrifuges. The relatively small sample size may limit the generalizability of the findings to larger populations or different clinical settings. Secondly, the study focused primarily on quantitative hematological parameters such as platelet and leukocyte counts, without assessing the functional quality or growth factor concentration of the PRP, which was not the study endpoint. Minor variations in handling time, temperature, and operator technique during centrifugation could have influenced platelet recovery and purity, especially hemoglobin contamination, despite attempts at standardization. Only two commercial centrifuge systems were evaluated, excluding other available devices which might be considered in future studies. Another serious limitation is that platelet count was calculated in the entire supernatant fluid rather than lower one third layer after either single or double spin in standardized centrifuges.

Although it is a limitation but also provides useful information to the users to never use the entire supernatant fluid just to increase the volume of PRP from a small specimen of blood or to decrease the time and cost of kits in order to do a proper technique. Our study does however show a sharp decline in platelet count in routine centrifuge specimens in all groups in the whole supernatant fluid, which is very concerning as in such a situation even the lower one third of layer might not yield any significant quantity of platelets. This calls for standardization of individual centrifuges and kits so as to yield maximum platelet concentrate for functional use. We intend to perform a future research on various spin speeds and cycles with different methods used for collection of fluid for second spin and checking only the lower third of the supernatant fluid, in an attempt to better describe viable options of PRP production from routine centrifuges. A wider range of commercial centrifuges can be compared both for efficiency of quantive production as well as qualitative outcome in various conditions.

## CONCLUSION

This study demonstrated that the method of centrifugation significantly influences the quality and composition of PRP. Commercially standardized centrifuge systems, particularly Neo PRP and Arthrex ACP, produced PRP with a significant rise in platelet counts, higher recovery rates, but slightly higher leukocyte and hemoglobin contamination which can be human error as well. However, platelet count markedly decreased in routine centrifuges, which likely reflects the use of entire supernatant fluid rather than lower one third in our study. Although most physicians and surgeons do use lower one third of supernatant fluid in their practice, many use the entire supernatant fluid as otherwise the volume of PRP will decrease from a single 10 cc specimen and more than one PRP kit might be needed. This discrepancy may lead to questionable results of PRP being used made from routine centrifuges. We recommend the use of lower one third of supernatant fluid using a double spin method while using routine centrifuges. Further studies are needed on various spin protocols as well as the use of only supernatant fluid in second spin cycle. Commercial standardized centrifuges on the other hand consistently produce reliable PRP concentrates in therapeutic range if the manufacturer recommendations are met, making it a more reliable, aseptic, single spin and contamination free method as in Arthrex ACP closed double syringe system. Standardization of the routine centrifuge systems may decrease the cost incurred on commercial centrifuges and kits in most situations.

### Authors’ contribution:

**SKA:** did concept design, manuscript writing, critical review of manuscript, statistical analysis and responsible for the accuracy of the study.

**AAH:** did data collection, manuscript writing and statistical analysis.

**SS:** did concept design, data collection and critical review of manuscript.
